# Letting people flourish: defining and suggesting skills for maintaining and improving positive health

**DOI:** 10.3389/fpubh.2023.1224470

**Published:** 2023-10-12

**Authors:** Hanne C. S. Sponselee, Lies ter Beek, Carry M. Renders, Willemieke Kroeze, Mirjam P. Fransen, Kristel M. van Asselt, Ingrid H. M. Steenhuis

**Affiliations:** ^1^Department of Health Sciences, Faculty of Sciences, VU University Amsterdam and Amsterdam Public Health Research Institute, Amsterdam, Netherlands; ^2^Care for Nutrition and Health Group, School of Nursing, Christian University of Applied Sciences, Ede, Netherlands; ^3^Department of Public and Occupational Health, Amsterdam UMC, Amsterdam, Netherlands; ^4^Department of General Practice, Amsterdam UMC, University of Amsterdam, Amsterdam, Netherlands

**Keywords:** positive health, health literacy, health promotion interventions, low socioeconomic position, skills for positive health

## Abstract

**Background:**

The concept of “positive health” emerged from the need for a holistic and more dynamic perspective on health, emphasising the ability of individuals to adapt and self-manage. The positive health conversation tool helps understand how people score on six positive health dimensions. However, skills within these dimensions to maintain or improve health have not yet been described. This is important for enabling individuals to put health advice into practise. Therefore, this paper aims to define and suggest skills for maintaining and improving positive health.

**Subsections:**

Suggestions for definitions of skills within the positive health dimensions are described using the functional, interactive, and critical health literacy framework. Additionally, executive functions and life skills were incorporated. Moreover, the environment's role in these individual skills was noted, mentioning organisational health literacy that emphasises organisations' responsibility to provide comprehensible health information to all individuals. We propose that health promotion interventions can incorporate the proposed skills in practical exercises while aligning intervention materials and implementation tools with end-users and implementers.

**Discussion and conclusion:**

The suggested skills for maintaining and improving positive health are a first step towards a more comprehensive understanding and open to discussion. These skills may also be applied to other practical conversation tools for maintaining or improving health. Increasing positive health through the defined skills may be especially relevant to those with a lower socioeconomic position who also have limited health literacy and thereby may contribute to reducing health inequalities. Taken together, strengthening the defined skills may hopefully contribute to allowing people to flourish in life.

## Background

In 1948, the World Health Organisation (WHO) defined health as “*a state of complete physical, mental and social well-being and not merely the absence of disease or infirmity”* (1). This definition was innovative because it considered the mental and social aspects of health next to the physical aspect for the first time. However, the use of the word “complete” is suggested to make health achievement rather unfeasible for most people and would thereby imply that most people are mostly unhealthy (2). Since the introduction of the WHO definition, almost three-quarters of a century has passed, and a need to move towards a more dynamic approach to health in research and practise has emerged. This need was reflected in the concept of “positive health”, developed in 2011 by Huber and colleagues, which describes health as “*the ability to adapt and to self-manage, in the face of social, physical and emotional challenges”* (2). The concept was further operationalized in 2016, after a shift in the Netherlands from a welfare state to a participation society in 2013, where the latter emphasised the need for individuals' adaptability and self-management (3). The further operationalization of positive health was based on a mixed-methods study among 140 stakeholders, such as healthcare professionals, patients with chronic diseases, and policymakers (4). Finally, positive health addressed six dimensions: bodily functions, mental functions and perception, spirituality or existentialism, quality of life, social and societal participation, and daily functioning (4). Moreover, the Handbook of Positive Health was developed, including a conversational tool with which a health worker and client can evaluate the client's condition in each dimension, considering what the client wants to maintain or improve and what requires guidance (4). Several studies have applied positive health, for example in health education and healthcare (5–8). However, specific skills within the six positive health dimensions needed to maintain or improve one's health have not yet been described. Defining these skills is relevant and critical to guide health workers in helping individuals practise received health advice by subsequently adapting and self-managing their health.

It is essential to build on existing concepts when defining these skills, such as health literacy, perceived as a crucial determinant influencing people's health. For example, a widely used definition of health literacy by Sørensen et al. (9) is “*people's knowledge, motivation, and competencies to access, understand, appraise, and apply health information in order to make judgements and take decisions in everyday life concerning healthcare, disease prevention, and health promotion”*. This definition emphasises that knowledge and motivation related to understanding and applying health information are vital yet insufficient when the skills to apply this knowledge and motivation are lacking. Furthermore, the definition of skills for maintaining and improving positive health should build on existing taxonomies and evidence for behaviour change techniques. These techniques are systematic practises integrated into behaviour change interventions as key elements (10, 11). Lastly, it should be emphasised that health literacy and interrelated skills should be viewed as strongly related on a continuum rather than as separate concepts.

Maintaining and improving health by strengthening skills to adapt and self-manage health may benefit a wide range of individuals; however, it is imperative to focus on individuals who would benefit most. People with a low socioeconomic position (SEP) have a relatively low healthy life expectancy (i.e. living in good health for a shorter amount of time) compared to those with a high SEP (12, 13). Many people with a low SEP also have relatively limited health literacy levels (14–16). In the Netherlands, it has been suggested that a quarter of adults are insufficiently health literate, and among people with a low SEP, 38% have been estimated as having insufficient health literacy levels (17). On behalf of the working group on positive health literacy at the Amsterdam Public Health Research Institute, this perspective article aims to propose ways forward in defining skills within the six positive health dimensions contributing to the maintenance and improvement of individuals' health by answering the following questions:

1. Which skills within the six positive health dimensions do individuals need to maintain or improve their health?
2. How can these skills specifically be improved among people with a low SEP who also have limited health literacy levels?

## 1. Which skills within the six positive health dimensions do individuals need to maintain or improve their health?

Before discussing the skills within the positive health dimensions that individuals need to maintain or improve their health, we should clarify the terminology regarding skills because they are occasionally mistaken for competencies. Skills are approached as specific abilities needed to achieve a goal. They are part of the broader concept of competencies, referring to a broader set of characteristics that include knowledge, thought patterns, and mindsets (18).

Different approaches to define skills within the six positive health dimensions are possible. We begin by defining skills for maintaining and improving positive health based on the health literacy concept because it is an important determinant of health (19) and includes previously defined skills. Below, we elaborate on these concepts and associated skills related to positive health.

### Health literacy and skills

Using health literacy as a starting point to define skills for maintaining and improving positive health can help by taking a closer look at health literacy skills. A systematic review in 2020 summarised 34 studies between 2000 and 2017 that provided a definition or detailed explanation of health literacy (20). Thus, combining the definitions conceptualising health literacy in terms of skills with the commonly used health literacy levels by Nutbeam in 2000 (21) may be a helpful starting point to define skills within the positive health dimensions to maintain and improve individuals' health:

- Functional health literacy (i.e. basic reading and writing skills). Basic literacy and numeracy skills have also been defined as part of health literacy in subsequent definition studies (22–26).
- Interactive health literacy (i.e. advanced skills for extracting information and deducing significance from various communication forms). Other health literacy definitions describe related advanced personal and social skills, such as confidence (e.g. asking for clarification of health information to ensure comprehension) (20), self-efficacy (i.e. participating in health care through acquired knowledge and understanding about one's health) (27), and communication skills (26, 28). They are also specified as application skills (i.e. skills to adhere to instructions or use procedures) (26).
- Critical health literacy (i.e. critical analysis skills, using information to be more in control in life). Related skills have been specified as information skills (i.e. accessing, understanding and managing health information, and critically analysing its credibility) (28), judgement skills (i.e. making judgements based on explicit knowledge that can be verbalised) (29) and management skills (e.g. comparing options) (30).

Based on the above, Table 1 describes suggestions for definitions of skills that can be achieved within each positive health dimension. The table was set up by combining the three-level health literacy definition by Nutbeam (21) with the six positive health dimensions and related aspects (4).

**Table 1:** Suggestions for definitions of skills within the six positive health dimensions to maintain and improve health.

**Positive health dimension**	**Dimensions' related aspects (4)**	**Proposed definitions of skills**				
		**Health literacy**			**Executive functioning**	**Life skills**
		**Functional health literacy** *Being able to…*	**Interactive health literacy** *Being able to…*	**Critical health literacy** *Being able to…*	*Being able to… (for example)* • Inhibition …inhibit the tendency to snack after dinner to adhere to a preferred eating pattern	*Being able to… (for example)* • Persistence …persist in having a job in order to derive meaning from it
Bodily functions	Medical facts, medical observations, physical functioning, complaints and pain, energy	Example situation: Having a consult with a life coach				
		… read information about lifestyle behaviour to prepare for a consultation with a life coach	… have a conversation with a life coach about health and prevention to formulate goals in lifestyle behaviours	… judge whether the life coach's advice applies in new circumstances to self-manage health	• Planning …create a plan with concrete objectives to adhere to a weekly physical activity goal	• Conscientiousness …consider important decisions carefully to be in charge of your life
Mental functions and perceptions	Cognitive functioning, emotional state, esteem/self-respect, experiencing being in charge/manageability, self-management, resilience, sense of coherence	Example situation: Verbalising feelings			• Shifting …flexibly adapt to new social environments to experience meaningful interactions	• Emotional stability …respond calmly to a socially challenging situation to reflect well on your emotions
		…write down how you feel to express an emotional state	…adhere to habits you know will benefit your mental state to improve mental well-being	…judge whether you should seek help from a professional to improve mental functions	• Updating …oversee your daily activities to self-manage desired changes for improved energy	• Optimism …approach the future with confidence to improve mental well-being
Spiritual/existential	Meaning/meaningfulness, striving for aims/ideals, future prospects, acceptance	Example situation: Integrating gratitude in everyday life			• Self-reflection …self-reflect on what your job means to you to potentially increase meaning	
		…write down three things you are grateful for that day to increase a sense of meaningfulness	…share something you have written in your gratitude journal with a relative to reflect on things meaningful to you	…assess if you derive enough meaning from your life by reflecting on your gratitude journaling to potentially derive more meaning		• Self-control …delay immediate gratification of an alcoholic drink to stick to the long-term goal of an alcohol-free month
Quality of life	Quality of life/well-being, experiencing happiness, enjoyment, perceived health, flourishing, zest for life, balance	Example situation: Reflecting on perceived health				
		…fill in questionnaires about quality of life to find out how you score	…verbalise how you experience your health during a conversation to reflect on your perceived health	…assess relevant health information on its credibility to determine how you can improve your perceived health		• Assertiveness …communicate your opinion towards a relative respectfully to maintain an equal relationship
Social and societal participation	Social and communicative skills, meaningful relationships, social contacts, experiencing to be accepted, community involvement, meaningful work	Example situation: Being in contact with relatives				• Refusal …reject a request for help to avoid crossing your limits
		…read an invitation to a wedding of a relative to feel involved	…actively listen to a relative and asking related questions to maintain a meaningful relationship	…reflect on your relative's perspectives to determine your personal view and improve social skills		• Relaxation …relax after a stressful conversation to restore your energy levels
Daily functioning	Basic activities of daily living, instrumental activities of daily living, ability to work, health literacy	Example situation: Making a daily schedule				
		…write down your desired daily activities in a calendar to be in charge of your daily life	…ask for help when more activities are planned than you can perform to cope with pressure from daily activities	…determine and control the priority of several daily activities to improve daily functioning		

### Executive functioning and life skills

**Figure 1:**
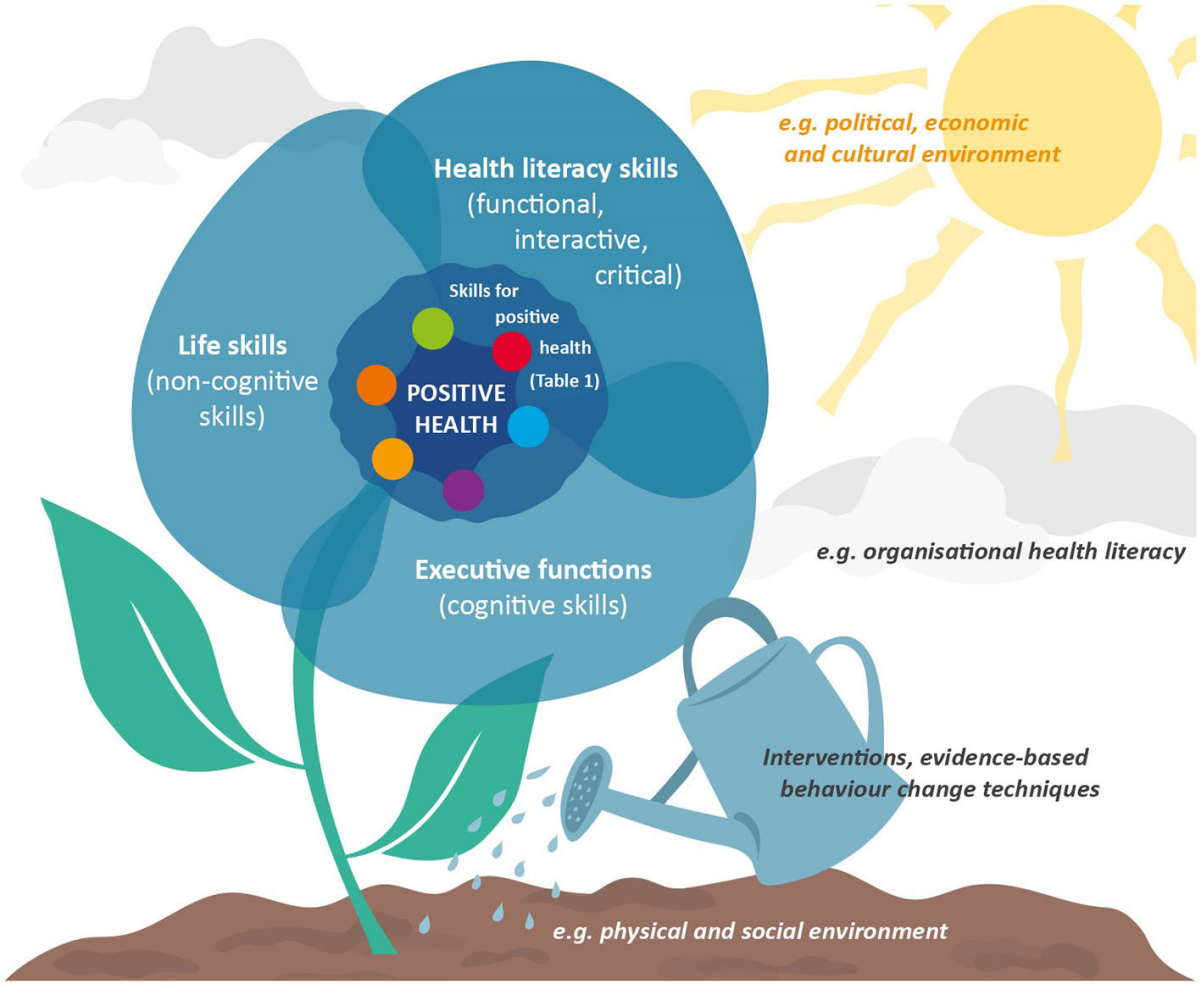
Cultivating skills for maintaining and improving positive health. The ecological system theory as defined by Bronfenbrenner (31) was used for this illustration.

Besides health literacy, people's health is also influenced by other individual factors, such as executive functioning and life skills (Table 1 and Figure 1). Executive functioning is defined as a range of high-order cognitive processes, with four main aspects: inhibition (conscious control over reactions), planning, shifting (cognitive flexibility), and updating (control over working memory) (32, 33). The cognitive process of self-reflection is also essential as a basic skill to improve health (34). Executive functioning enables individuals to organise, plan, and execute goal-oriented and purposeful behaviour (35, 36). Moreover, a review concluded that executive functioning is a critical construct in maintaining health and is related to various underlying mechanisms of health (37). For example, poor executive functioning has been consistently linked to overweight and obesity (38) through a lack of impulsivity control and increased odds of overeating (39, 40). Because of the overall link between these cognitive processes and health, we propose that the aforementioned executive functions (i.e. inhibition, planning, shifting, updating, and self-reflection) should be considered skills for maintaining and improving positive health.

Furthermore, life skills play a role in individuals' health. Life skills are mouldable personal characteristics referred to as “non-cognitive” compared to cognitive skills (e.g. executive functioning) (41). Steptoe and Wardle (41) showed that accumulating the five life skills of persistence, conscientiousness, emotional stability, optimism, and control may result in beneficial outcomes such as self-rated health, economic success, and social function. Another study examined the relationship between life skills (operationalized as self-control, assertiveness, refusal and relaxation) and showed a positive relationship with resilience (42). Resilience is also described in the positive health dimension “mental functions and perception”. Overall, we suggest that the aforementioned non-cognitive life skills (i.e. persistence, conscientiousness, emotional stability, optimism, self-control, assertiveness, refusal, and relaxation) should also be considered for maintaining and improving positive health.

#### *Organisational health literacy as an important factor* 

Beyond individual cognitive and non-cognitive factors, environmental factors such as organisational health literacy play an overarching role in individuals' health. Individuals interact with a complex web of environments (31), so supporting and strengthening positive health should focus on them (7). Indeed, many environments involve complicated health information landscapes, with most health information in written form. Although individuals' basic literacy remains crucial in these complex environments, organisations are responsible for facilitating navigation through their information. Organisational health literacy (OHL) captures this need, described as the extent to which organisations “*make it easier for people to navigate, understand, and use information and services to take care of their health”* (43). Stakeholders at organisational levels, such as community workers and healthcare professionals, are responsible for providing health information to facilitate individuals obtaining and navigating through the information initially. These stakeholders surrounding individuals within organisations are essential for improving individual health status (44). Health literacy competencies (e.g. professional skills such as conversing and consulting) of healthcare personnel are, for example, classified under OHL (45), as their competencies may indirectly influence individuals' health literacy levels (46–49). Thus, OHL seems to be a vital environmental aspect for consideration with skills for maintaining and improving positive health (Figure 1). In addition, the extent to which individuals are encouraged by organisations and health professionals to take control themselves instead of immediately offering help is important to empower individuals to self-manage health.

## 2. How can these skills specifically be improved among people with a low socioeconomic position who also have limited health literacy levels?

Maintaining and improving skills for positive health is important for everyone, although the potential to increase health is probably relatively high for people with a low SEP who also have limited health literacy levels. People with a low SEP often face multiple short-term problems (e.g. financial problems) (50), which can leave little cognitive space to engage in long-term health behaviour change (44, 51). Therefore, it is crucial to support people with a low SEP in addressing and coping with these problems and, if possible, help resolve them. The subsequent cognitive space may be an enabling factor in having more opportunities to engage in health behaviours. Besides, it is essential to give people with a low SEP the opportunity to gain a better grip on their health. Furthermore, limited health literacy has been related to adverse mental health outcomes (e.g. loneliness, social isolation) (52–54), unhealthy eating behaviour and physical inactivity (16), poorer take-up of preventive services (55), and low general literacy levels (55, 56). Taken together, people with a low SEP who also have limited health literacy levels may benefit most from improving the skills for maintaining and improving positive health. Here, it is important to emphasise that people do not need to improve all the skills listed. It is especially important to first identify all relevant skills that affect health, then discuss with clients which skills they want to work on and then determine on which skills it is meaningful and feasible to intervene. Thus, the skills to be worked on differ from individual to individual (57).

The Handbook of Positive Health states that the diversity of patients in the consulting room is increasing, creating increased complexity (58). This complexity requires a broader view and more time because a tailored approach is needed. The authors inquire, “*Are there special concerns about working with positive health in people with low socioeconomic status?”*. The most vulnerable people always benefit the least from any intervention. As individuals and their environment are inseparably linked (59), responsibility for health and lifestyle should not be placed entirely on the individual. Policy should be put in place to create a healthy environment, which also should facilitate and support that clients can properly learn and adopt the proposed skills. This could be done, for example, by addressing organisational health literacy and providing suitable health promotion interventions.

### Health promotion interventions to improve skills within the six positive health dimensions

Giving people with a low SEP who also have limited health literacy the opportunity to gain a better grip on their health can be achieved through enabling and facilitating participation in suitable interventions. Participation in health promotion interventions can be a good starting point for engaging in health behaviour change. Many health promotion interventions focusing on behaviour change among people with a low SEP exist, including improved dietary patterns, physical activity, and reduced smoking (60). Health literacy interventions for people with a low SEP often appear to focus on improving health literacy skills through, for example, group activities (e.g. role play) and goal setting (14). However, to our best knowledge, there has been no overview of concrete skills within the positive health dimensions used in interventions for people with a low SEP who also have limited health literacy levels.

Hence, it is imperative that the proposed skills are included in interventions in an accessible way and by using evidence-based behaviour change techniques to improve health in the six positive health dimensions. These skills should be incorporated into practical exercises with concrete and straightforward learning objectives to enable the monitoring of participants' progress. An example of a health promotion intervention that performed in this way is the Dutch *Gezond en goed met elkaar* (healthy and well together) intervention, which focuses on the simultaneous increase of positive health and general literacy levels among people with a low level of health literacy (61). Its development was co-created with people with a low level of health literacy and lifestyle coaches to implement the intervention based on evidence-based behaviour change techniques, such as motivational interviewing (62), formulating implementation intentions (63), goal setting (64), setting graded tasks (65) and planning coping responses (66). Preliminary qualitative results showed that participants valued participating because they felt less lonely (i.e. the positive health dimension of *mental functions and perception*) and more confident in day-to-day life (i.e. positive health dimension of *daily functioning*) (61). Beyond incorporating skills into practical exercises, increasing the suitability of interventions to the living worlds of people with a low SEP who also have limited health literacy may help increasing participation and intervention effectiveness (57, 67). Therefore, intervention materials and implementation tools must be aligned with prospective end-users and implementers to increase intervention suitability (68).

## Discussion and conclusion

Positive health offers a broad perspective, making it an interesting concept within public health because it focuses less on solving health problems and more on people's ability to adapt and self-manage physical, emotional, and social challenges. However, the positive health tool used in practise is currently aimed at what to achieve within the six positive health dimensions, not how to maintain and improve them. This perspective article aimed to define skills within the six positive health dimensions contributing to maintaining and improving individuals' health while suggesting how these skills can be improved among people with a low SEP who also have limited health literacy levels. We proposed specific skills by describing health literacy, executive functions, and life skills. It is essential to remember that the systems surrounding individuals influence health (Figure 1) and to include organisational health literacy. The defined skills may also be applicable to other practical conversation tools. Examples are the Dutch *Leefstijlroer* (lifestyle rudder) for healthcare professionals to discuss healthy lifestyles with clients (69), the *4Domeinen model* (4-domains model) for professionals in the medical and social fields to inventory life domains and perceived health with patients (70) and the CONNECT instrument with which a physician can assess patients' health condition (71). The suggested skills are a first step towards their more comprehensive definition, which, like the concept of health, is subject to change. Thus, we are open to receiving ideas about additions or adjustments to the defined skills.

Concerning positive health among people with a low SEP who also have limited health literacy levels, these individuals can benefit from help in solving and dealing with problems in various life areas possibly hindering health behaviour change. We proposed that health promotion interventions could incorporate the defined skills to improve health with practical exercises and learning objectives. Furthermore, intervention materials should be suitable for end-users, and implementation tools should be aligned with the implementers.

A possible limitation of this paper is that we have not conducted a systematic literature review, and this is therefore the first theoretical attempt of defining the skills. Future empirical research should show whether these defined and suggested skills are relevant in practise. Additionally, we recognise that difficult social conditions can impair health in individuals. Even though positive health can be a tool to work towards increased health, it does not release governments from their task to invest in social equality and circumstances. Individuals should not be blamed for adverse conditions due to a lack of skills for positive health.

Increasing the health of people with a low SEP who also have limited health literacy levels can contribute to individual health while contributing to the reduction of health inequalities. This reduction has proven insufficient over the years in Europe, despite various attempts in research, practise, and policy (72). Systems thinking, which considers multiple components of systems holistically (73), is needed to understand and address socioeconomic health inequalities (74). Several theoretical models have emphasised the importance of considering individual and environmental levels to reduce socioeconomic health inequalities (31, 59). Thus, there remains a way ahead to incorporate the suggestions of the defined skills for maintaining and improving positive health into components of systems surrounding individuals.

All in all, the defined and suggested skills within the six positive health dimensions can hopefully contribute to improved individual health while allowing people to flourish in life.

## Data availability statement

The original contributions presented in the study are included in the article/supplementary material, further inquiries can be directed to the corresponding author.

## Author contributions

LB and IS conceptualised the study. HS wrote the first draft of the manuscript and final draft of the manuscript. LB, CR, WK, MF, KA, and IS edited the text. All authors contributed to manuscript revision, read, and approved the submitted version.
